# The multi-target small-molecule inhibitor SB747651A shows in vitro and in vivo anticancer efficacy in glioblastomas

**DOI:** 10.1038/s41598-021-85536-4

**Published:** 2021-03-16

**Authors:** Arnon Møldrup Knudsen, Henning Bünsow Boldt, Elisabeth Victoria Jakobsen, Bjarne Winther Kristensen

**Affiliations:** 1 Department of Clinical Research, University of Southern Denmark, Odense, Denmark; 2 Department of Pathology, Odense University Hospital, J.B. Winsløws Vej 15, 5000 Odense, Denmark

**Keywords:** CNS cancer, Cancer models, Targeted therapies

## Abstract

Glioblastoma multiforme is the most common primary brain tumor and among the most lethal types of cancer. Several mono-target small molecule-inhibitors have been investigated as novel therapeutics, thus far with poor success. In this study we investigated the anticancer effects of SB747651A, a multi-target small-molecule inhibitor, in three well characterized patient-derived glioblastoma spheroid cultures and a murine orthotopic xenograft model. Concentrations of 5–10 µM SB747651A reduced cell proliferation, spheroid formation, migration and chemoresistance, while apoptotic cell death increased. Investigation of oncogenic kinase signaling showed decreased phosphorylation levels of mTOR, CREB, GSK3 and GYS1 leading to altered glycogen metabolism and formation of intracellular reactive oxygen species. Expression levels of cancer stemness marker SOX2 were reduced in treated tumor cells and SB747651A treatment significantly prolonged survival of mice with intracranial glioblastoma xenografts, while no adverse effects were observed in vivo at doses of 25 mg/kg administered 5 days/week for 8 weeks. These findings suggest that SB747651A has anticancer effects in glioblastoma. The cancer-related pathophysiological mechanisms targeted by SB747651A are shared among many types of cancer; however, an in-depth clarification of the mechanisms of action in cancer cells is important before further potential application of SB747651A as an anticancer agent can be considered.

## Introduction

Glioblastoma multiforme is the most common primary brain tumor and is among the most deadly cancers with a median survival of approximately 15 months, despite aggressive treatment efforts including maximum surgical resection followed by concomitant radio- and chemotherapy with the alkylating agent temozolomide (TMZ)^1^. Glioblastomas often harbor alterations in several signaling pathways involved in crucial elements of cancer pathogenesis, including regulation of cell growth, proliferation and survival mechanisms. Commonly altered pathways include PI3K-PTEN-Akt-mTOR, EGFR, RAS-MAPK and P53 signaling^2^, which are often deregulated in many different types of cancer^3–6^. The MAPK signaling pathway has been subject to development of many therapeutic approaches targeting EGFR, Ras, ERK, PI3K, mTOR and Akt^7–10^, with promising preclinical data, but limited results in clinical trials. Resistance to therapy is a major obstacle, partly because of compensating signaling mechanisms that bypass the inhibited target and partly because of significant tumor heterogeneity. Multi-targeted therapies are currently under investigation, and seem to be a promising strategy to combat acquired resistance and to improve treatment efficiency, but their full potential still remains to be elucidated^11,12^.

SB747651A, also known as 2-(4-Amino-1,2,5-oxadiazol-3-yl)-1-ethyl-*N*-4-piperidinyl-1*H*-imidazo[4,5-*c*]pyridine-7-methanamine dihydrochloride is an ATP-competitive small-molecule inhibitor that inhibits MSK1/2 and RSK1/2 in the MAPK pathway, and Akt as well as p70S6K in the PI3k-Akt-mTOR pathway. SB747651A thus has targets in both the classical ERK MAP kinase pathway, in the JNK/p38 MAP kinase pathway and in the PI3k-Akt-mTOR pathway, thereby simultaneously inhibiting multiple different pathways, and providing a robust framework for application of SB747651A as an anticancer agent. MSK1 has been implicated in resistance to PI3K/mTOR inhibitors in glioblastomas^13^ and is suggested to repress pro-apoptotic genes, thereby promoting cell survival^14^. RSK2 has been demonstrated to regulate glioblastoma motility and cell invasion, and RSK2 inhibition can sensitize cells to TMZ treatment and reduce cell proliferation^15,16^. The role of Akt in glioblastoma tumorigenesis has been well described^17,18^, and combined Akt and p70S6K inhibition has been shown to reduce migration and invasion^19^ and to inhibit glioblastoma xenograft growth^20^. Furthermore, p70S6K levels have been associated with response to chemotherapy in advanced ovarian cancer^21^. Based on this, we hypothesized that SB747651A may be used as an agent for treatment of glioblastoma, and potentially also other cancer types. The objective of this study was to investigate the anticancer effects of SB747651A using glioblastoma as a model system, by applying well characterized primary patient-derived glioblastoma spheroid cultures, and a murine orthotopic glioblastoma xenograft model.

## Materials and methods

### Cell cultures

The three patient-derived primary GBM spheroid cultures T78, T86, and T111 established in our laboratory between 2009 and 2012^22^ were cultured as free floating spheroids in serum-free medium (NBE) consisting of Neurobasal-A medium (Gibco) supplemented with 1% B27 (Invitrogen), 0,5% N2 (Invitrogen), 1% L-Glutamine (Gibco), 1% Penicillin + streptomycin (Gibco), 25 ng/ml EGF (Sigma-Aldrich), and 25 ng/ml bFGF (PeproTech). Cells were cultured in a humidified incubator at 36 °C with 5% CO_2_. All cell lines have previously been thoroughly characterized and validated by spheroid formation and differentiation assays, molecular subtyping, sequencing with the Oncomine Comprehensive Assay v3 and with a custom NGS panel for mutational status of 20 of the most frequently mutated genes in glioma^23^, Illumina 850 k methylation array and by orthotopic xenograft implantation in mice.

## Cell viability, LC50 and apoptosis assays

SB747651A and A1070722 were purchased from Tocris Bioscience. GBM spheroids were incubated with either NBE medium alone or NBE medium with 5 µM or 10 µM SB747651A for 1 day, 4 days, 7 days or 10 days in 12 well plates. Propidium Iodide (Sigma-Aldrich) and CellEvent Caspase-3/7 Green Detection Reagent (ThermoFisher Scientific) was added to a final concentration of 0.02 mM, and cells incubated for 3 h in the dark. Grayscale fluorescence images were acquired immediately thereafter with a Leica DM IRB inverted fluorescence microscope equipped with a Leica DFC300 FX camera. For LC50 experiments, cells were treated for 48 h with a tenfold dilution range from 1 mM to 1 nM SB747651A prior to addition of Propidium Iodide. The fluorescence intensity of individual spheroids was analyzed with the ImageJ software (V1.52a).

### Chemosensitivity assay

Tumor spheroids were dissociated into single cells with TrypLE (Gibco). After 5 days, when new spheroids had formed, the medium was replaced, and 200 μl cell suspension was added to each well in a 96-well plate. SB747651A and TMZ were added to the wells to make the following treament groups: 5 μM or 10 μM SB747651A combined with 30 μM, 60 μM, 120 μM or 240 μM TMZ. The spheroids were incubated for 48 h, before Propidium Iodide was added, and images acquired and analyzed as previously described. Each exposure condition was performed with 6 technical replicates, and at least 25 spheroids were measured per group per replicate experiment.

### Limiting dilution assays and measurements of spheroid growth

Tumor spheroids were dissociated into single cells, and re-suspended in NBE, NBE + 5 uM SB747651A, or NBE + 10 uM SB747651A. A serial dilution was made using three 96-well plates per group with the following cell numbers seeded in 12 wells per plate: 10,000/well, 1,000/well, 100/well, 10/well, and 1/well. After incubation for 10 days, the fraction of wells not containing spheroids was counted, log-transformed, and plotted against the number of cells/well with the ELDA software^24^, thereby calculating the tumor-initiating-cell frequency (TICF). For measurement of spheroid diameters, three random images at X10 magnification were acquired per well-plate of the wells with 10,000 cells/well.

### Migration assay

A serum-free flat surface migration assay was used, as previously described^25^. Briefly, Geltrex (Gibco) was mixed with NBE medium (1:50), and 1.4 ml was added to each well in 12-well plates. The coated well-plates were incubated overnight at 36 °C and the supernatant aspired. A single GBM spheroid was seeded in each well, and well-plates were incubated for 90 min at 36 °C, prior to addition of 1 ml medium to each well.

For migration assays with SB747651A, spheroids were pre-treated with 5 or 10 µM SB747651A for 24 h. Images were acquired immediately after spheroid seeding, and regularly every 24 h for a total of 3 days. Cell death in migrating cells was investigated by addition of Propidium Iodide to the wells after 72 h of migration. The migration distance was measured with the ImageJ software.

### Phospho-protein antibody arrays

The Proteome profiler human phospho-MAPK array kit (R&D Systems) was used to measure relative changes in phosphorylation levels of 26 different MAPK according to manufacturer’s instructions. Briefly, samples were prepared by seeding 3 million T78 cells into T150 flasks and then leaving the cells to recover for 24 h, before 10 µM SB747651A was added and left to incubate for additional 24 h. Cells were then spun down at 100 RCF for 7 min at 4 ^°^C, washed with ice-cold PBS twice, and lysed with the included kit lysis buffer supplemented with Halt protease and phosphatase inhibitor cocktail (ThermoFisher Scientific) at 2X final concentration, and stored at -80 °C until use. Protein concentrations in the lysates were determined with the Pierce BCA protein assay kit and 200 μg total protein lysate was used per membrane. CL-XPosure x-ray film was exposed for 3 min, digitalized, and the images were analyzed with ImageJ software.

### Glycogen metabolism assays

Cell lysates were prepared from T78 cells cultured in NBE and cells exposed to 10 μM SB747651A for 72 h were spun down at 100 RCF for 7 min at 4 °C, washed with ice-cold PBS twice, and lysed with ddH_2_O at 8 × 10^6^ cells/ml. Lysates were heated to 100 °C for 10 min, spun down at 14,000 RCF for 5 min and the supernatant was pipetted into a new Eppendorf tube and stored at − 80 °C Glycogen concentrations were measured in the samples with the Glycogen Assay Kit II (Colorimetric) (Abcam) according to manufacturer’s instructions using 96-well plates and a BioTek EL808 plate reader with absorbance measurements performed at 450 nm.

### SB747651A in vivo toxicity assessment

Female balb/c nude mice aged 7–8 weeks (n = 11), were divided into three groups: A vehicle (HBSS) treated control group (n = 3) and 2 treatment groups receiving either 5 mg/kg (n = 4) or 25 mg/kg (n = 4) intraperitoneal injections of SB747651A. Since no pharmacokinetic or pharmacodynamics data for SB747651A was available, administered doses were estimated based on dosage regimens of other small molecule inhibitors in preclinical studies^26–28^. An initial single administration was performed and the animals observed closely for 7 days. Subsequently, the animals were treated 5 days/week for 4 weeks total, with close monitoring of body weight and behavior. Animals were then euthanized, and the liver, kidneys and brain from each animal was formaldehyde fixed and paraffin-embedded. A dose of 25 mg/kg was used in the orthotopic xenograft model.

### Orthotopic glioblastoma xenograft model

Orthotopic glioblastoma xenografts were implanted as previously described^29^. Briefly, female balb/c nude mice aged 7–8 weeks (n = 36) were anaesthetized and placed in a stereotactic frame. A small skin incision was made on the skull, bregma was located and periost removed. A small burr-hole was made 1 mm anterior and 2 mm laterally from bregma using a dental drill. A 2 µl suspension of 300,000 T78 tumor cells was slowly injected into the right hemisphere with a Hamilton syringe at a depth of 3 mm. The syringe was slowly retracted, and the skin was sutured. Immediately after tumor cell implantation, the mice were block randomized (1 cage = 1 block) into four groups with the Research Randomizer tool (30): Vehicle treated control group (n = 9), SB747651A 25 mg/kg for 8 weeks (n = 9), TMZ 30 mg/kg for 5 days (n = 9) and SB747651A 25 mg/kg daily for 8 weeks + TMZ 30 mg/kg for 5 days (n = 9). All animals were monitored on a daily basis and euthanized if they displayed any sign of neurological deficit or lost ≥ 20% body weight. A predefined endpoint of 250 days was chosen for termination of the experiment in case of no signs of tumor burden. Organs were removed and processed post-mortem as previously described. From each animal, a blood sample was taken with a 300 µl Microvette CB serum tube (Sarstedt), and spun down at 2000 RCF for 5 min. The serum was isolated and frozen immediately at − 80 ˚C.

### Alanine transaminase activity and creatinine measurements

Serum Alanine transaminase (ALT) and creatinine levels were measured with the colorimetric ALT activity assay and Creatinine assay kit (Sigma-Aldrich) in 96-well plates according to manufacturer’s instructions. Each sample was assayed as technical duplicates and 10 µl serum was used per well. Absorbance was measured at 562 nm on a BioTek EL808 plate reader.

### Immunohistochemistry and automated digital quantification

Tumor spheroids were immersion-fixed in 4% neutral-buffered formaldehyde, paraffin-embedded, and cut into 3 μm sections on a microtome. Sections were deparaffinized followed by blocking of peroxidase activity. After heat-induced-epitope retrieval, sections were stained with Hematoxylin/Eosin and the following antibodies: Nestin (Clone: 196,908, 1:1000, R&D Systems), Bmi-1 (Clone: F6, 1:200, Upstate Biotechnology), CD133 (Clone: W6B3C1, 1:40, Miltenyi Biotec), SOX2 (Clone: 245,610, 1:200, R&D Systems), Musashi-1 (Clone: 14H1, 1:200, MBL International Corporation), phospho-GSK3 (Clone: EPR933Y, 1:100, Abcam), phospho-GYS1 (Ser641) (Clone: PA5-17,702, 1:400, ThermoFisher scientific), phospho-CREB (Ser133) (Clone: 87G3, 1 + 800, Cell Signaling Technology), and phospho-mTOR (Clone: 49F9, 1 + 200, Cell Signaling Technology).

The paraffin-embedded mouse organs were stained with Hematoxylin/Eosin and Periodic Acid-Shiff. Additionally, the brains of tumor-bearing animals were stained with human-specific Vimentin (Clone: SP20, 1:600, NeoMarkers) to identify tumor cells. The Vimentin stainings were performed on the Dako Autostainer Link 48 platform (Dako), while all other stainings were performed on the Ventana Discovery Ultra staining platform (Ventana Medical Systems).

Stained slides were digitalized using the NanoZoomer 2.0HT digital image scanner (Hamamatsu photonics, Japan) and imported into the microarray module of the Visiopharm software (V2018.9.4). Two different classifiers were programmed; One for detection of positive and negative nuclei, and one for detection of positive cytoplasmic staining. The fractions and mean intensitites of positive cells and staining regions were quantified with the software.

### Statistical analysis

Statistical analyses were performed with the GraphPad Prism software (V8.3.1). In datasets without normal distribution, multiple group comparison was performed with Kruskal–Wallis tests supplemented with Dunn´s multiple comparison tests. Normal-distributed datasets were analyzed with one-way ANOVA and Tukey´s posttests. Migration assays were analyzed with 2-way ANOVA and subsequent Bonferroni posttests. Comparison of two groups was performed with students t-test. *P*-values < 0.05 were considered statistically significant, and where possible, two-tailed tests were used. Error-bars represent mean ± SEM. Each experiment was performed as at least three independent biological replicates and the exact number of replicates for each experiment is stated in the corresponding figure legend.

### Ethical approval

This study was approved by the Danish Data Inspection Authority (approval Number 16/11065) and the Regional Scientific Ethical Committee of the Region of Southern Denmark (approval Number S-20150148). Animal experiments were approved by The Animal Experiments Inspectorate in Denmark (approval Number 2018–15-0201–01471) and reported in compliance with the ARRIVE guidelines. All experimental procedures in this study, including use of patient material and data, have been performed according to local and national guidelines and regulations, covered by permissions from the Danish Data Inspection Authority (approval Number 16/11065) and the Regional Scientific Ethical Committee of the Region of Southern Denmark (approval Number S-20150148).

## Results

### SB747651A induces apoptosis-mediated cell death and shows additive effects on cell death when combined with the alkylating chemotherapeutic agent temozolomide

**Figure 1:**
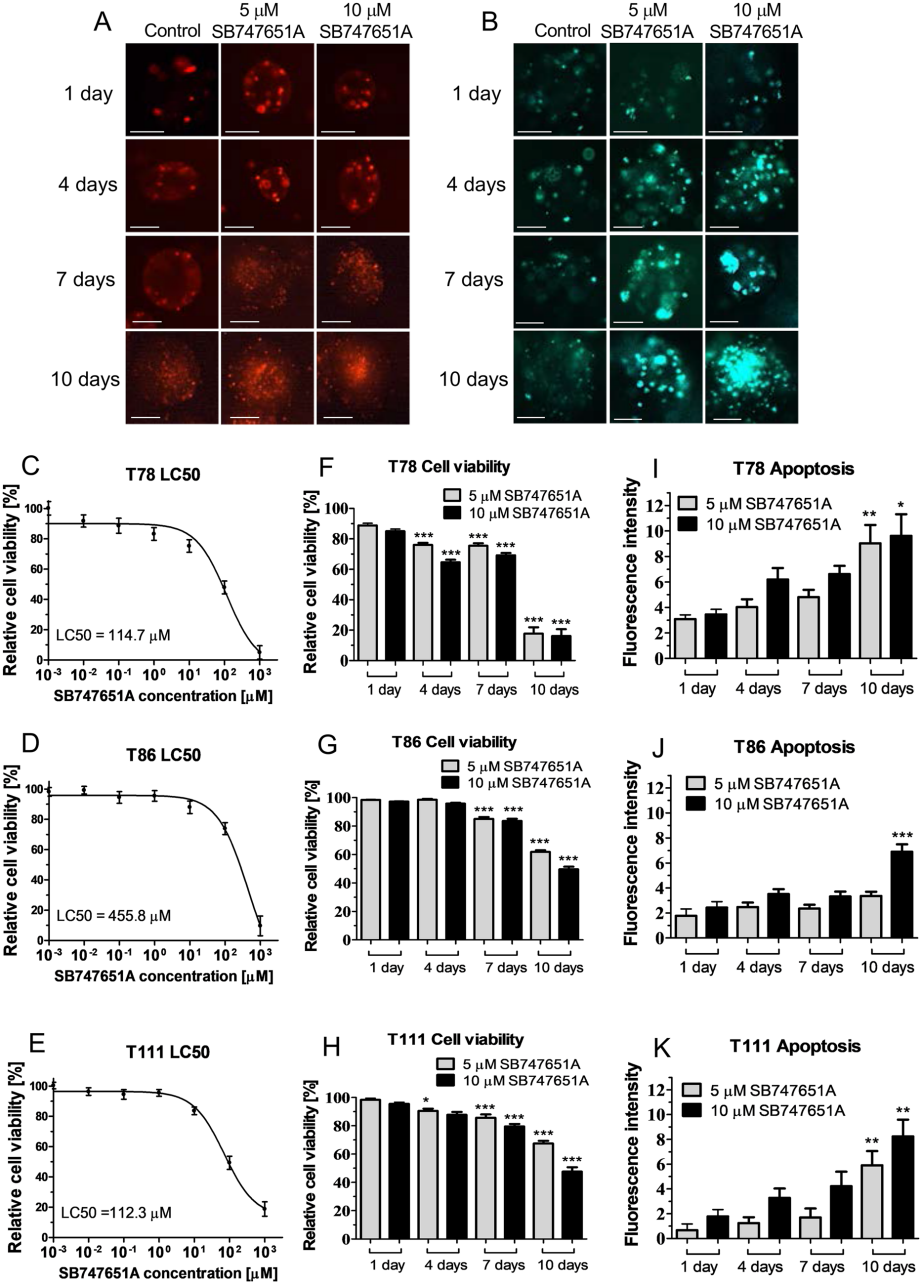
Cell viability and apoptosis following SB747651A exposure. (**A–B**) Representative images of Propidium Iodide (red) and Caspase 3/7 (green) fluorescence in the T111 spheroid culture after exposure to 5 or 10 μM SB747651A for 1, 4, 7 and 10 days respectively. (**C–E**) LC50 values showed different sensitivities to SB747651A exposure, with the T78 and T111 spheroid cultures showing the lowest LC50 values. (**F–K**) Relative cell viability decreased and Caspase 3/7 fluorescence intensity increased in a concentration- and time-dependent manner in all three spheroid cultures. Displayed significance levels are all compared to the fluorescence intensity of the corresponding measurement at 24 h. Fluorescence intensity is shown as arbitrary units. * = *P* < 0.05. ** = *P* < 0.01. *** = *P* < 0.001. Scale bar for images from 1 and 4 days = 100 μm. Scale bar for images from 7 and 10 days = 200 µm. Data shown are pooled from 3 independent experiments.

We found a significant time- and concentration-dependent increase in Propidium Iodide and Caspase 3/7 fluorescence intensities in all three spheroid cultures with maximum intensities reached after 10 days exposure (Fig. 1A–B). After 48 h treatment duration, the T111 spheroid culture showed the lowest LC50 value of 112.3 μM followed by the T78 spheroid culture with an LC50 of 114.7 μM. T86 cells had a substantially higher LC50 value of 455.8 μM (Fig. 1C–E). When comparing the 5 and 10 μM exposure groups, we found significant differences in relative cell viability and Caspase 3/7 fluorescence intensities, with the highest concentration showing the highest cell death and apoptosis in all spheroid cultures (Fig. 1F–K). SB747651A exposure induced apoptosis-mediated cell death, which increased with both concentration and exposure time. LC50 values for SB747651A treatment drastically decreased when increasing the exposure duration; The T78 spheroid culture had LC50 values of 123.3 and 96.1 µM after 4 and 7 days exposure respectively (supplementary Fig. 1A), the T86 cells had LC50 values of 62.1 and 20.1 µM after 4 and 7 days (supplementary Fig. 1B), while the T111 cells had LC50 values of 92.2 and 5.38 µM after 4 and 7 days SB747651A exposure (supplementary Fig. 1C).

Next, cells were exposed to different combinations of SB747651A and TMZ. We found an increase in cell death that positively correlated with TMZ concentration (supplementary Fig. 2A). Addition of either 5 or 10 μM SB747651A in combination with TMZ resulted in an additive concentration-dependent increase of cell death. This effect was most pronounced in the T78 and T111 spheroid cultures (supplementary Fig. 2B+D), which have methylated O(6)-Methylguanine-DNA methyltransferase (MGMT) promoters. The T86 spheroid culture is MGMT-promoter unmethylated, and showed high resistance to both TMZ and TMZ + SB747651A combinations (supplementary Fig. 2). In general, all three spheroid cultures showed high resistance to TMZ treatment, with LC50 values of 370.7 µM for T78 cells, 564.9 µM for T86 cells, and 273.9 µM for T111 cells after 48 h TMZ treatment (supplementary Fig. 2E–G).

### SB747651A inhibits spheroid formation and impairs spheroid growth

**Figure 2:**
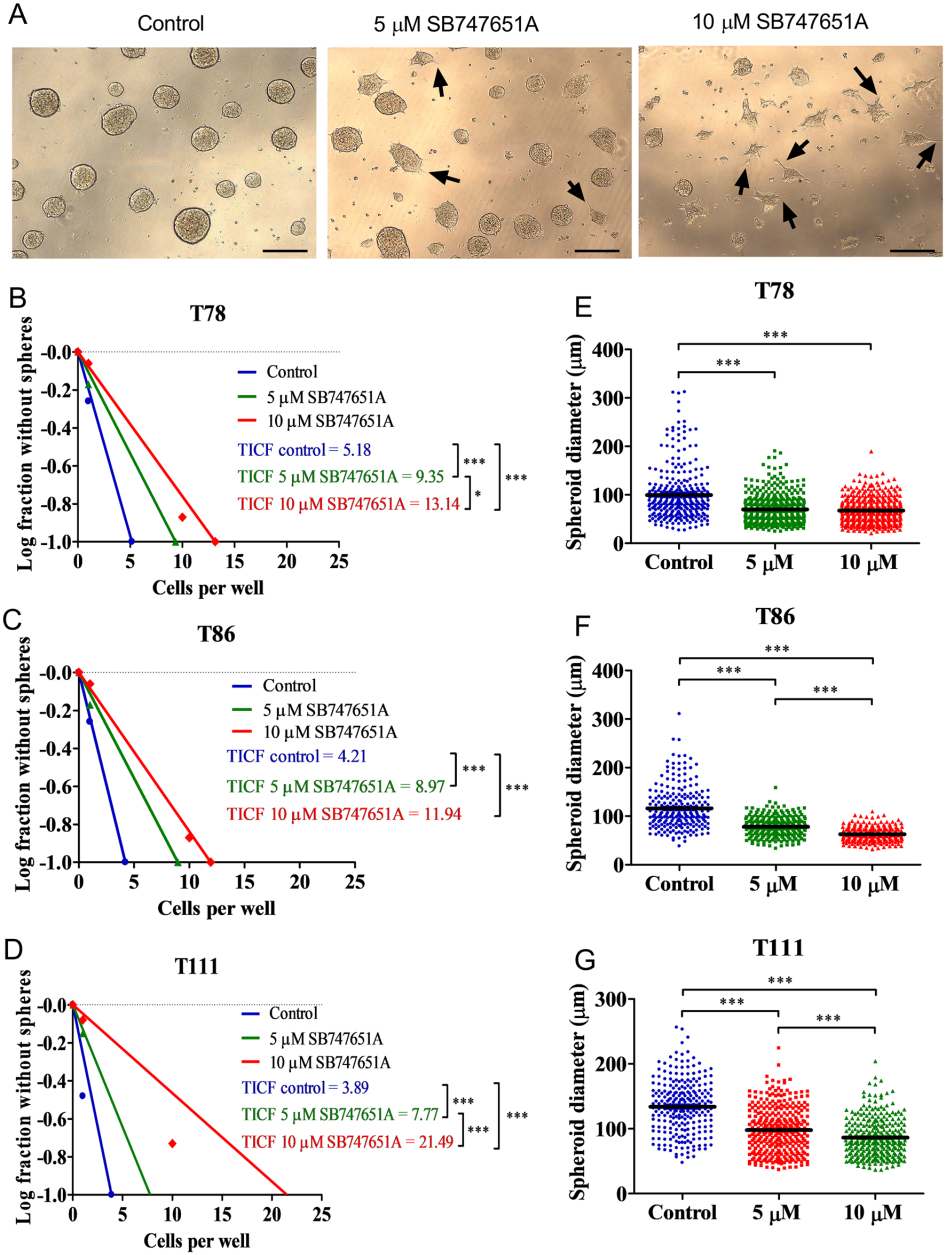
Limiting dilution assays and spheroid growth after SB747651A treatment. (**A**) Representative images of spheroids from the T111 spheroid cultures from limiting dilution assays. The black arrows indicate pseudopod-like extensions from spheroids exposed to SB747651A. Scale bar = 200 μm. (**B–D**) Following SB747651A exposure, the tumor-initiating-cell frequency was significantly reduced in all three spheroid cultures in a concentration-dependent manner. (**E–G**) The diameter of spheroids exposed to SB747651A was significantly smaller than controls. Furthermore, this effect was concentration-dependent in the T86 and T111 spheroid cultures. * = *P* < 0.05. ** = *P* < 0.01. *** = *P* < 0.001. Data shown are pooled from 3 independent experiments.

The impact of SB747651A exposure on tumor spheroid formation was investigated with limiting dilution assays. Interestingly, we observed morphological changes of the spheroids in the SB747651A exposure groups which appeared smaller and had non-circular appearances with pseudopod formations (Fig. 2A). After 10 days exposure, we found that both the 5 and 10 µM SB747651A groups had a significantly lower frequency of spheroid formation, compared to the vehicle control group in a concentration-dependent manner across all spheroid cultures (Fig. 2B–D). The T78 cells had a tumor-initiating-cell-frequency (TICF) of 1 in 5.18 cells (95% CI 4.20–6.39), which was reduced to 1 in 9.35 cells (95% CI 7.47–11.71) in the 5 µM group, and 1 in 13.14 cells (95% CI 10.32–16.74) in the 10 µM group (Fig. 2B). The TICF of T86 cells was 1 in 4.21 (95% CI 3.41–5.21), 1 in 8.97 cells (95% CI 7.17–11.21) in the 5 µM group and 1 in 11.94 cells (95% CI 9.42–15.14) in the 10 µM group (Fig. 2C). For T111, the TICF was 1 in 3.89 cells (95% CI 3.14–4.82), 1 in 7.77 cells (95% CI 6.25–9.66) in the 5 µM group and 1 in 21.49 cells (95% CI 17.52–26.38) in the 10 µM group (Fig. 2D). All groups had the ability to form spheroids at clonal density, but with significantly decreasing frequencies in the SB747651A exposure groups.

The diameter of spheroids from the limiting dilution assays was measured and compared across all groups to investigate whether SB747651A also affected spheroid growth. In the T78 spheroid culture, treated spheroids were significantly smaller compared to controls (control mean = 99.42 µm, 5 µM mean = 69.99 µm, 10 µM mean = 67.58 µm, *P* < 0.001, Fig. 2E). Similar results were found for T86 (control mean = 116.05 µm, 5 µM mean = 78.23 µm, 10 µM mean = 62.97 µm, *P* < 0.001, Fig. 2F) and T111 spheroid cultures (control mean = 133.97 µm, 5 µM mean = 97.82 µm, 10 µM mean = 86.28 µm, *P* < 0.001, Fig. 2G). In the T86 and T111 spheroid cultures the 5 µM and 10 µM groups furthermore differed significantly from each other (*P* < 0.001), demonstrating a concentration-dependent response.

**Figure 3:**
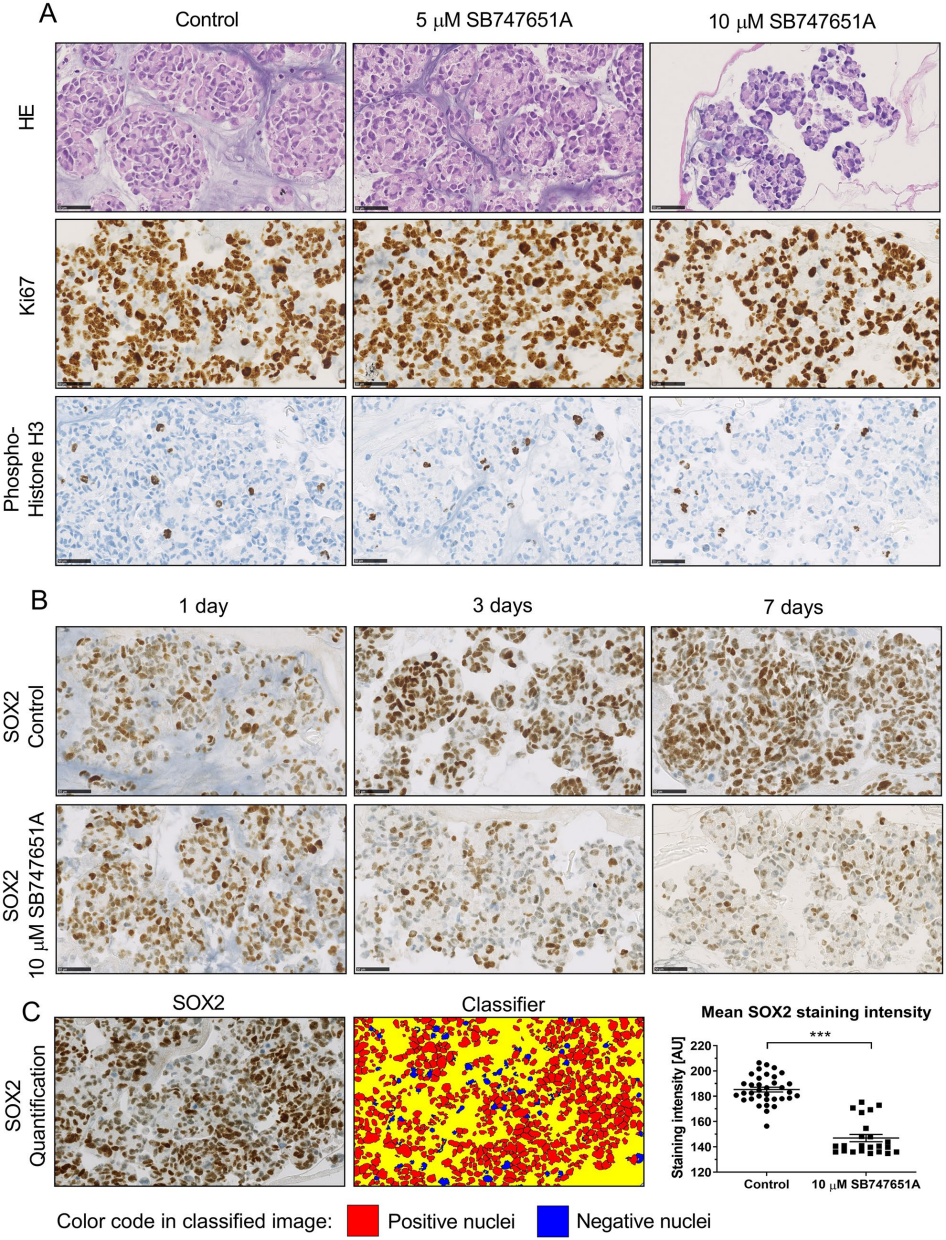
Immunohistochemical stainings of tumor spheroids. Representative images from the T78 spheroid culture taken at 20X magnification. Scale bar = 50 µm. (**A**) The Ki-67 proliferation index was approximately 90% and similar across all exposure groups, as was expression of phospho-Histone H3. (**B**) A decrease of SOX2 expression was observed after exposure to 10 µM SB747651A in a time-dependent manner. (**C**) Representative images of the software-based digital quantification of SOX2 stainings. The classifier was programmed to identify positive nuclei (red) and negative nuclei (blue)). Quantification was performed on 4 independent experiments with 3 days of SB747651A exposure. AU = arbitrary units. * = *P* < 0.05.

Based on this, we hypothesized that exposure to SB747651A could reduce cell proliferation and induce cellular differentiation. This was further investigated by immunohistochemical stainings of paraffin-embedded tumor spheroids. Stainings for Ki-67 showed a very high proliferation rate, and stainings for phospho-Histone H3 (Ser10), being a marker for ongoing mitosis, showed no notable differences in amount of labeled nuclei (Fig. 3A). Stainings for several stemness related markers revealed a significant decrease of SOX2 staining intensity in SB747651A exposed tumor spheroids (mean intensity 149.5 arbitrary units) vs. controls (mean intensity 187.4) (*P* = 0.02, Fig. 3B–C). Expression of Nestin, Bmi1, CD133 and Musashi-1 were unchanged in exposure groups compared to controls (supplementary Fig. 3), indicating a specific inhibition of SOX2 expression.

### SB747651A reduces glioblastoma cell migration

**Figure 4:**
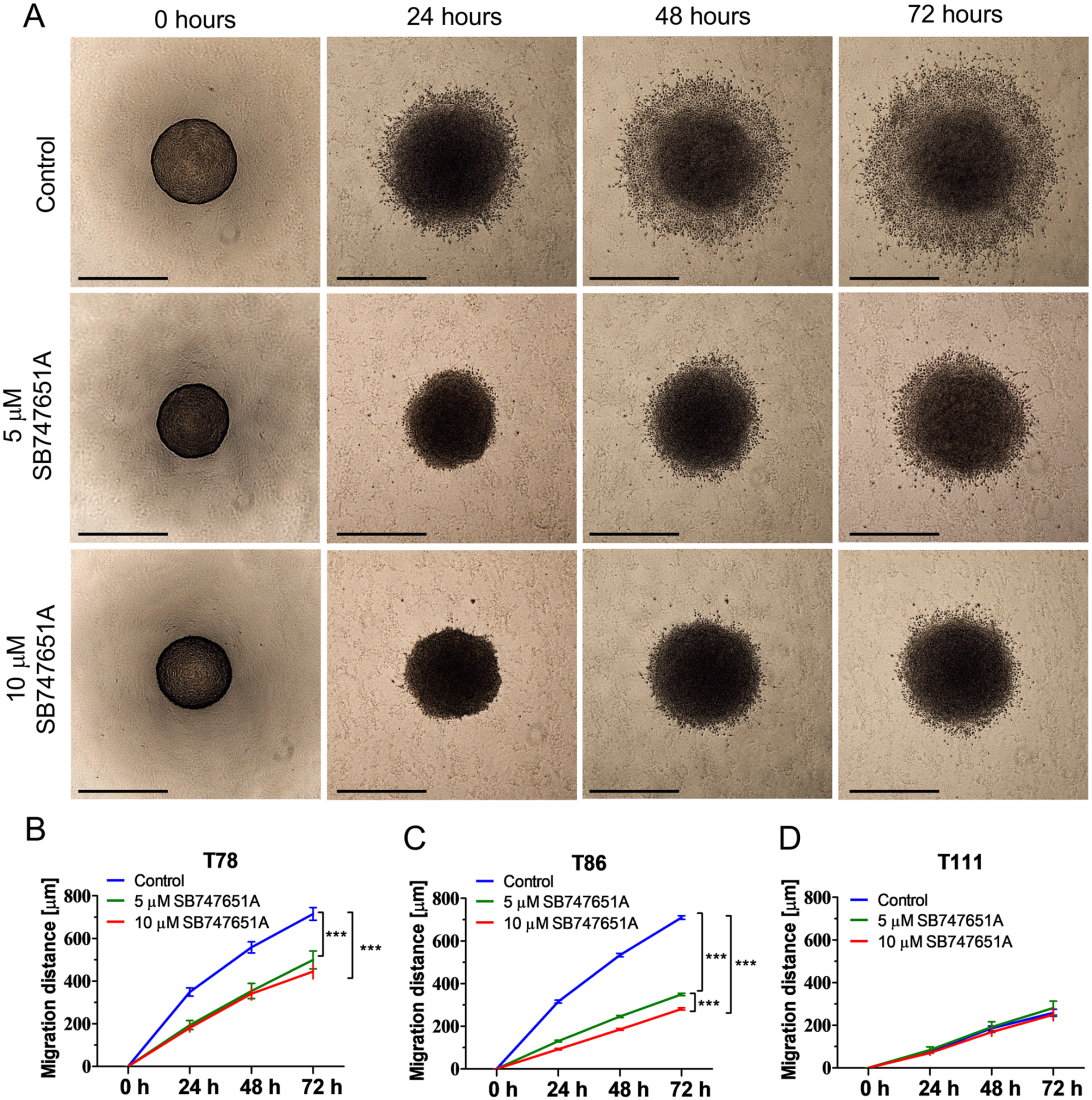
Cell migration assays. (**A**) Representative images of spheroids from the T86 spheroid culture used in the serum-free migration assay. (**B–C**) Spheroids from the T78 and T86 spheroid cultures exposed to SB747651A showed significantly impaired cell migration when compared to controls, and for T86 this effect was furthermore concentration-dependent. (**D**) Spheroids from the T111 spheroid culture migrated very poorly, and no difference in cell migration distance was observed. *** = *P* < 0.001. Scale bar = 500 μm. Data shown are pooled from 3 independent experiments.

Next, we investigated the effect of SB747651A on cell migration (Fig. 4A).SB747651A exposed T78 spheroid cultures showed a reduction in migration distance by 30.2% in the 5 µM group and 37.8% in the 10 µM group (*P* < 0.001, Fig. 4B). In the T86 spheroid culture, the migration distance was reduced by 50.8% in the 5 µM group and 60.4% in the 10 µM group (*P* < 0.001, Fig. 4C). In the T111 spheroid culture there was no difference in migration distance between treated and control groups (Fig. 4D). Addition of Propidium Iodide to migrating cells after 72 h of migration showed that migrating cells exposed to SB747651A were still viable, indicating that SB747651A inhibits cell migration in a non-lethal manner (supplementary Fig. 3B).

### SB747651A reduces phosphorylation levels of several MAP-kinases and alters glycogen metabolism

**Figure 5:**
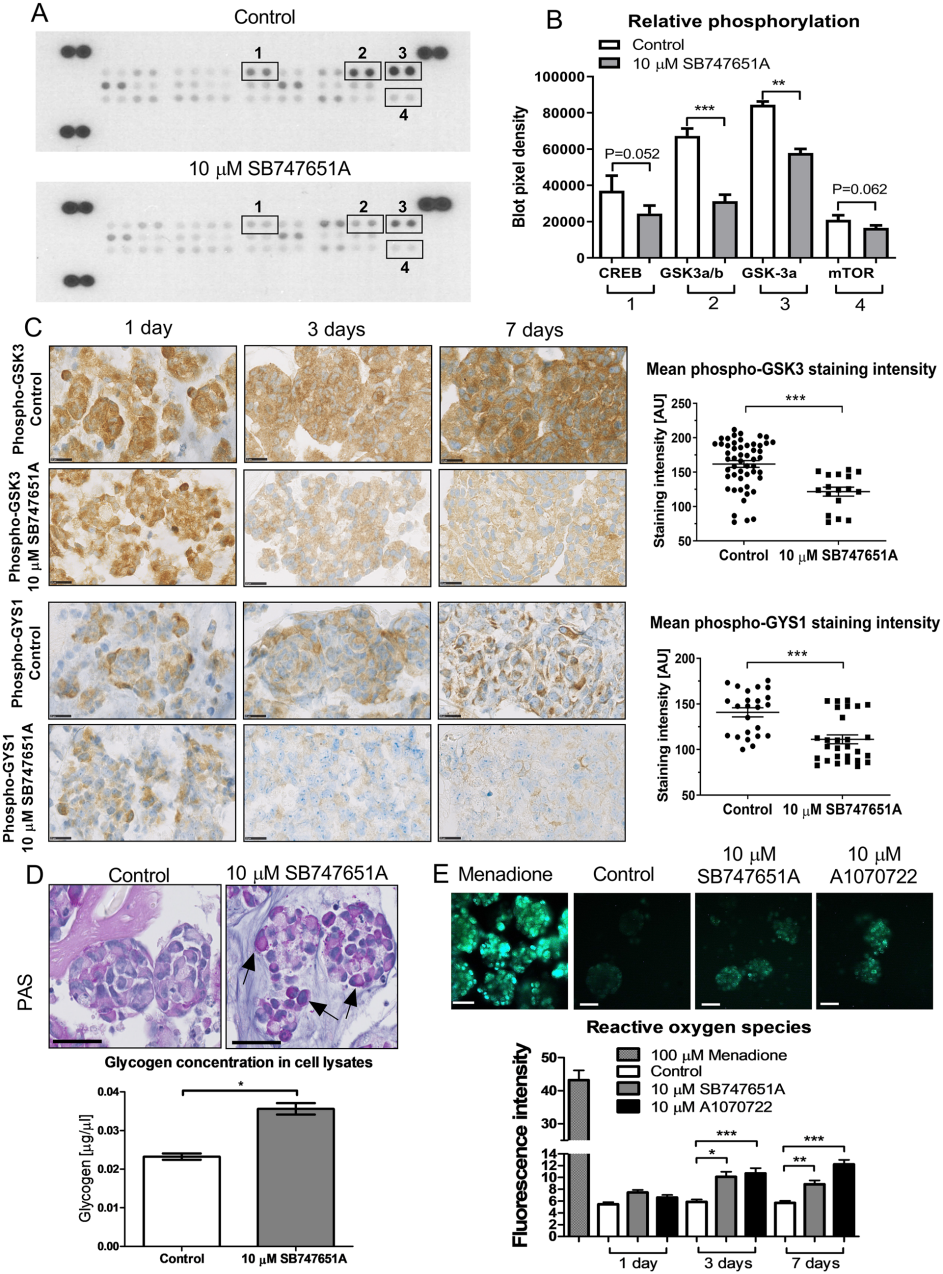
SB747651A exposure alters MAPK phosphorylation and modulates glycogen metabolism. (**A**) Representative images of two separate membranes from the Proteome profiler Phospho-MAPK assay. Each membrane was loaded with 200 µg total protein lysate and exposed on X-ray film for 180 s. Membranes are depicted in their entirety without cropping, and assay control dots are seen in the corners of each membrane. Comparisons of control vs. treated cells were made on separate membranes, which were all processed and analyzed identically at the same time. (**B**) Quantification of 4 independent experiments showed a significant reduction in GSK3α/β phosphorylation levels, and borderline significant reductions of phospho-CREB and phospho-mTOR. (**C**) Reductions in phosphorylation levels of GSK3 and its downstream substrate, the GYS1 kinase, were confirmed by immunohistochemistry and software-based digital quantification performed on T78 tumor spheroids. Scalebar = 50 µm. (**D**) Periodic Acid Schiff staining of SB747651A treated spheroids showed an intracellular accumulation of glycoproteins (black arrows). Scale bar = 50 µm. (**E**) SB747651A and A1070722 treated spheroids showed lower expression of phospho-GSK3 protein and intracellular ROS formation compared to controls. Menadione was used as a positive control for ROS induction. * = *P* < 0.05. ** = *P* < 0.01. *** = *P* < 0.001.

To identify the mechanisms of action of SB747651A treatment, we investigated relative phosphorylation levels of 26 different phospho-proteins involved in MAPK signaling (Fig. 5A). We found a significant reduction in phosphorylation levels of GSK3α/β (53.4% reduction, *P* < 0.001), and a borderline significant reduction in CREB (34% reduction, P = 0.052) and mTOR phosphorylation (21.4% reduction, *P* = 0.062) (Fig. 5B) after 48 h exposure to 10 µM SB747651A. Additionally, we observed reduced phosphorylation of RSK1 S380 (7.8% reduction), RSK2 S386 (9.1% reduction), MSK2 S360 (22.1% reduction) and p70S6K T421/S424 (20.3% reduction), however due to substantial variation between sample replicates, these results were not significant (supplementary Fig. 4). Surprisingly, no changes were found in Akt phosphorylation levels.

**Figure 6:**
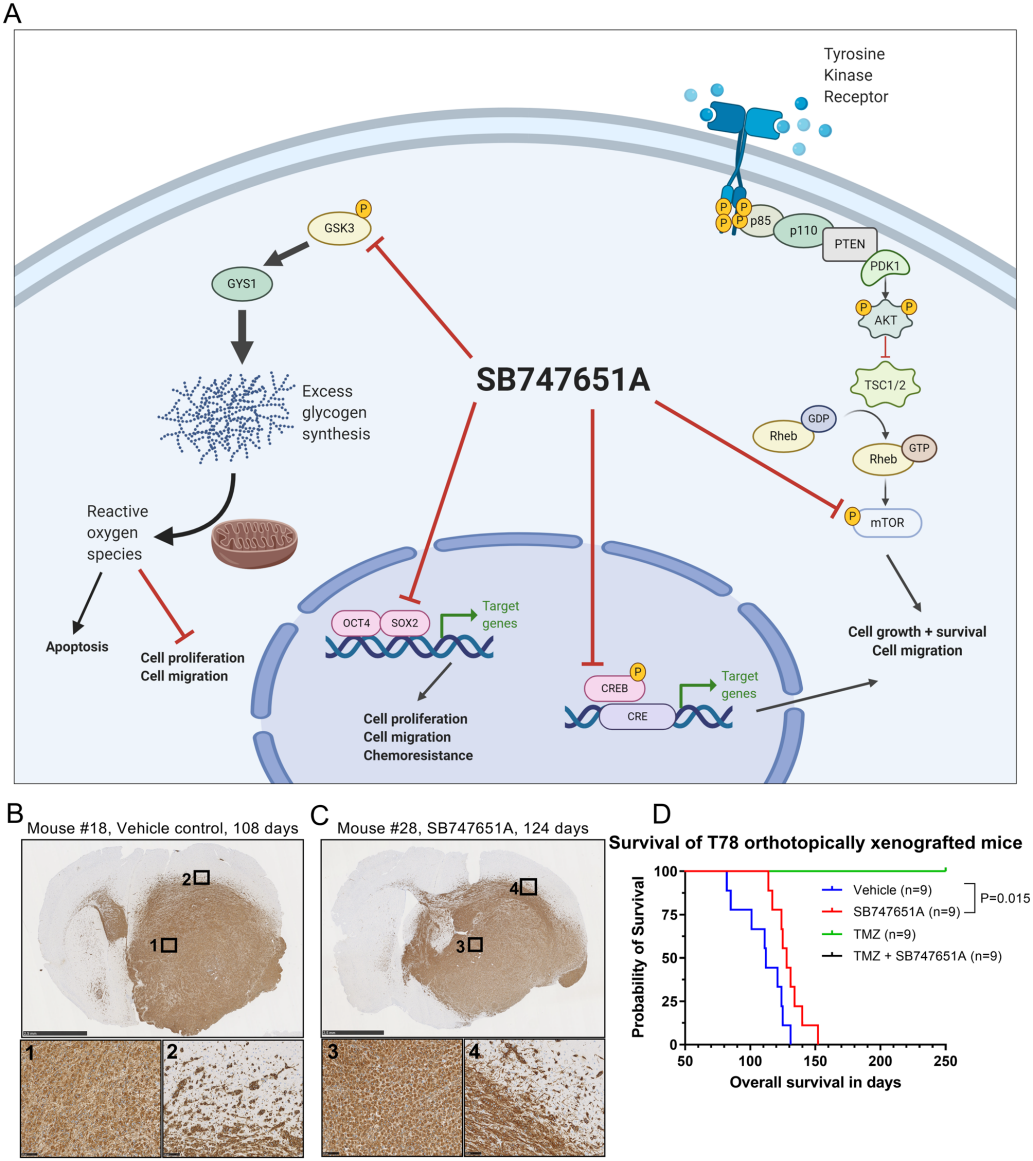
Schematic overview of SB747651A and in vivo orthotopic xenograft model. (**A**) Schematic illustration of intracellular targets and mechanistic functions of SB747651A. **(B–C**) Representative images of coronal brain sections from mice implanted with T78 orthotopic xenografts and treated with vehicle or SB747651A (n = 9 per group). Overview images were acquired at 1.25X magnification with scale bars = 2.5 mm. Inserts were acquired at 20X magnification with scale bars = 100 µm. Note that the midline shift in the vehicle treated mouse is substantially more pronounced than in the SB747651A treated mouse. (**D**) Kaplan–Meier survival curves for mice in the different treatment groups.

These changes in relative phosphorylation levels following 10 µM SB747651A exposure identified on the membrane blots were further validated by immunohistochemistry, and we could confirm a significant reduction of both phospho-GSK3 staining intensity (36.9% reduction, *P* = 0.001, Fig. 5C) and its downstream substrate, the phospho-GYS1 kinase (31.4% reduction, *P* = 0.001, Fig. 5C). The significant downregulation of phospho-CREB and phospho-mTOR was also confirmed immunohistochemically (supplementary Fig. 5). The fraction of phospho-CREB positive cells was reduced from 71.4% in control spheroids to 21.7% in SB747651A treated spheroids (*P* = 0.001, supplementary Fig. 4A–B), while the staining intensity of phosho-mTOR in exposed spheroids was reduced by 31.1% following 10 µM SB747651A treatment (*P* = 0.001, supplementary Fig. 5C–D). GSK3 and GYS1 are major regulators of glycogen synthesis, and a reduction in GSK3 phosphorylation reduces the inhibitory phosphorylation of GYS1. This results in a net-increase of GYS1 activity, thereby leading to increased synthesis of glycogen. To investigate whether this occurred in SB747651A exposed spheroids, we performed Periodic Acid Schiff stainings on T78 spheroids, and measured intracellular glycogen concentrations in cell lysates. SB747651A treated spheroids accumulated more intracellular glycoproteins compared to controls (Fig. 5D), and indeed, the intracellular concentration of glycogen was significantly higher in SB747651A treated spheroids compared to controls (*P* = 0.03). Accumulation of excess glycogen induces mitochondrial formation of reactive oxygen species (ROS), and we could confirm that SB747651A treated spheroids showed significantly higher levels of ROS compared to controls (Fig. 5E). This finding was reproduced using the GSK3 inhibitor A1070722, thereby linking the increased ROS formation to a decrease in GSK3-phosphorylation levels. Phospho-GSK3 protein expression levels was 60.6% of controls in 10 µM SB747651A treated spheroids and 50.9% of controls in 10 µM A1070722 treated spheroids. The proposed mechanistic function of SB747651A is schematically outlined in Fig. 6A.

### SB747651A treatment prolongs overall survival of orthotopically xenografted mice and shows no adverse effects in vivo

Next, we investigated the effect of SB747651A treatment on overall survival in a murine orthotopic xenograft model using the T78 spheroid culture (Fig. 6B–C). Mice in the SB747651A group (n = 9) had a significantly longer median survival of 128 days compared to 112 days in the vehicle group (n = 9; *P* = 0.015, Fig. 6D). No mice in the TMZ (n = 9) or TMZ + SB747651A (n = 9) treatment groups had tumors at the predefined endpoint of 250 days. No signs of acute lethal toxicity, weight loss or behavioral changes were observed in treated mice after initial single administration or after 4 weeks of continuous treatment with 25 mg/kg SB747651A for 5 days/week (Supplementary Fig. 6A). Creatinine levels and alanine transaminase activity did not differ between SB747651A and vehicle treated animals (Supplementary Fig. 6B–C). Histological examination of liver, kidney and brain tissue showed no signs of pathological changes in SB747651A treated animals (Supplementary Fig. 6D).

## Discussion

In this study we have investigated the effect of the small-molecule inhibitor SB747651A on several key elements in glioblastoma maintenance and progression. To our knowledge, the only published study investigating SB747651A in cancer, found that 20 μM SB747651A reduced invasion of oral squamous cell carcinoma in vitro, but had no effect on apoptosis or cell growth^31^. We found that patient-derived glioblastoma spheroid cultures subject to short-term SB747651A exposure showed only modest cell death and apoptosis with very high LC50 values > 100 µM, which most likely results in highly unspecific target inhibition. When extending exposure durations with 5–10 µM SB747651A to 7 and 10 days, the effect on cell death and apoptosis drastically increased, and LC50 values simultaneously decreased with increasing exposure durations, suggesting that maximum effect requires long-term exposure, and that short-term exposure is not a feasible approach for profound induction of cell death and apoptosis.

We found that SB747651A reduces phosphorylation levels of GSK3, GYS1, mTOR and CREB. In glioblastomas, GSK3 inhibition has previously been shown to induce apoptosis^32^, differentiation and reduce proliferation by affecting spheroid formation capabilities^33^. Additionally, GSK3 inhibition has been shown to attenuate migration/invasion in vitro and in vivo^34^, and to reverse TMZ resistance and sensitize glioblastoma cells to TMZ in vitro^35,36^. High GSK3 levels have furthermore been correlated with poor patient survival, and GSK3 inhibition in combination with TMZ has increased patient survival compared to TMZ alone^37^.

GYS1 inhibition, in primary glioblastoma cells has been shown to induce cellular accumulation of glycogen, which has been associated with a reduction of proliferation and migration, and an increase of cellular differentiation^38^. We were able to show that GSK3 and GYS1 inhibition by SB747651A prompts formation of ROS, which has been linked to induction of apoptosis^39^ and a decrease in stemness, self-renewal capabilities and mTOR activity in glioma stem-like cells^40^. The combined inhibition of GSK3/GYS1 and mTOR therefore acts synergistically to enhance the effects of SB747651A. The reduction of SOX2 expression we found following SB747651A exposure could be linked to the decreased fraction of treated tumor cells with spheroid formation abilities, since SOX2 is a crucial stemness and self-renewal protein in glioblastomas. Interestingly, the T111 spheroid culture was most susceptible to a decline in self-renewal capabilities after SB747651A exposure, which could be explained by a lower baseline level of stemness markers, thereby making it more susceptible to a decrease in SOX2 expression. SOX2 downregulation has previously been associated with a reduction in proliferation, invasion, tumorigenicity and chemoresistance^41–43^, while high SOX2 levels have been associated with poor patient prognosis^43^. We found a consistent decrease in phosphorylation of mTOR and CREB after SB747651A exposure, and since the pro-tumorigenic role of mTOR in glioblastomas has been well established^9,44^, mTOR inhibition is likely a partial functional mechanism of SB747651A. CREB is a downstream effector kinase in both the MAPK and PI3K pathways, and converges several cellular pathways. In glioblastomas, CREB has been shown to regulate growth, proliferation and differentiation^45,46^. Some of the targeted pathophysiological mechanisms have previously been uncovered as important glioblastoma drivers. However, SB747651A´s multi-targeting of glioblastoma metabolism, mediated by GSK3 and GYS1, combined with mTOR and CREB inhibition, and a decrease of SOX2 expression, has not previously been described. To specifically determine the effects of each target protein, knockout cells could be applied to investigate specific effects mediated by individual target proteins to uncover contributing protein functions. Single-target inhibitors blocking mTOR, GSK3 and CREB are commercially available. However, single-target mTOR inhibitors such as Temsirolimus, Sirolimus and Everolimus have been disappointing in clinical trials, and currently dual mTOR inhibitors such as Vistusertib are under investigation^47^. Monotarget GSK3β inhibition is currently under clinical investigation (NCT03678883), while CREB inhibition is under preclinical safety investigation^48^. Multi-kinase inhibitors, such as Sunitinib, are emerging in clinical investigations^49^ (NCT03025893), demonstrating that multi-kinase inhibitos can indeed be applied for treatment of patients, with acceptable and tolerable adverse effects.

No behavioral, biochemical or histological changes were observed in vivo in tumor bearing mice at doses of 25 mg/kg SB747651A administered for 8 weeks, suggesting very good tolerability throughtout the treatment duration. SB747651A treated animals survived significantly longer compared to vehicle treated animals, thereby demonstrating anticancer efficacy of SB747651A. The blood–brain-barrier penetration of SB747651A has not been addressed in this study, and should be elucidated in future work to gain insights into central nervous system bioavailability and pharmacokinetics. The treatment duration in our study only accounted for less than half of the overall survival time of treated mice, and it can therefore be speculated that the effect of SB747651A could be even more pronounced if treatment duration is extended beyond 8 weeks. The absence of adverse effects would make this a feasible approach to potentially further prolong overall survival.

No animals in the TMZ or TMZ + SB747651A groups had tumors at day 250, indicating that TMZ alone was sufficient to eradicate all tumor cells, which may be explained by the relatively rapid initiation of TMZ treatment starting 2 weeks after tumor cell implantation. The additive effect of TMZ + SB747651A we found in vitro could therefore not be evaluated in vivo*.* However, a potential additive effect cannot be excluded based on our results, and this aspect should be investigated in future studies.

Taken together, the multi-kinase inhibitor SB747651A has shown a series of different anticancer effects in glioblastomas, which in combination have shown promising potential of the inhibitor as a drug for treatment of this devastating disease. The effects of SB747651A could very well extend to other cancer types beyond our findings in glioblastomas, as many of the relevant pathophysiological mechanisms are shared among different types of cancer. We provide some insights into the mechanism of action, however, prior to future investigations of SB747651A as an anticancer agent, the full spectrum of mechanisms of action should be clarified. Potential future studies should aim to elucidate the synergy of SB747651A combined with other treatment modalities such as TMZ and/or radiation therapy and to optimize treatment duration and dosage regimen. Particular emphasis should be directed towards advancing and expanding preclinical results, including direct comparison with existing PI3K/Akt/mTOR inhibitors.

## Supplementary information

Supplementary information.
