# Supplementary material for: The multi-target small-molecule inhibitor SB747651A shows in vitro and in vivo anticancer efficacy in glioblastomas
Source: Sci Rep. 2021 Mar 16;11:6066. doi: 10.1038/s41598-021-85536-4 (PMC7966768; doi:10.1038/s41598-021-85536-4)
Supplement: Supplementary file 1 — Supplementary information. [file 41598_2021_85536_MOESM1_ESM.pdf]

The multi-target small-molecule inhibitor SB747651A shows in vitro and in vivo anticancer efficacy in glioblastomas

Arnon Møldrup Knudsen (1,2), Henning Bünsow Boldt (1,2), Elisabeth Victoria Jakobsen (1,2), Bjarne Winther Kristensen (1,2).

1. Department of Clinical Research, University of Southern Denmark, Odense, Denmark.
2. Department of Pathology, Odense University Hospital, Odense, Denmark.

Supplementary figure 1

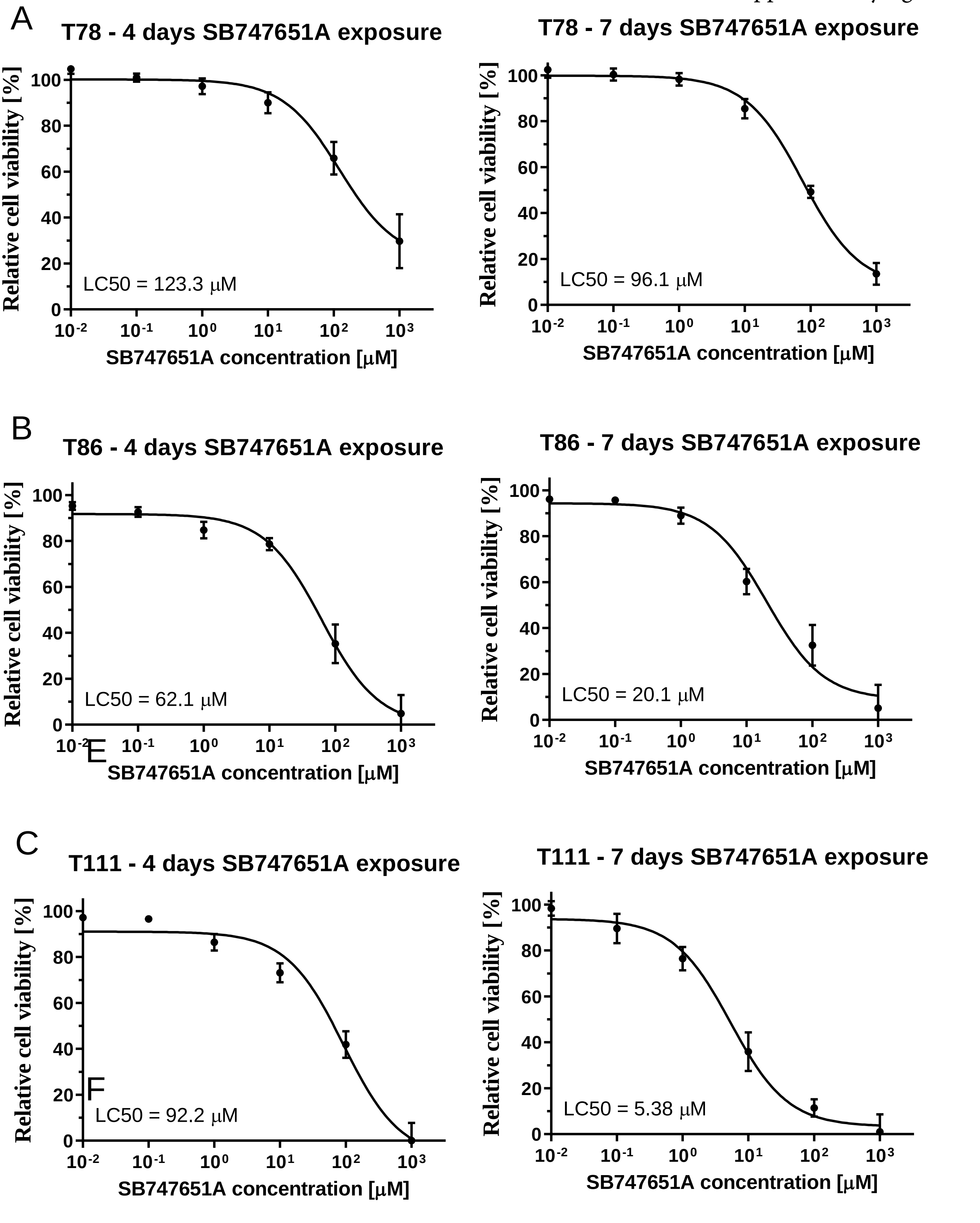

Supplementary figure 1. LC50 values following long-term SB747651A exposure.

LC50 values are graphically displayed for **A)** T78, **B)** T86 and **C)** T111 spheroid cultures after 4 and 7 days exposure to SB747651A respectively. Note the inverse relationship between exposure duration and LC50 values. Error-bars represent mean ± SEM.

Supplementary figure 2

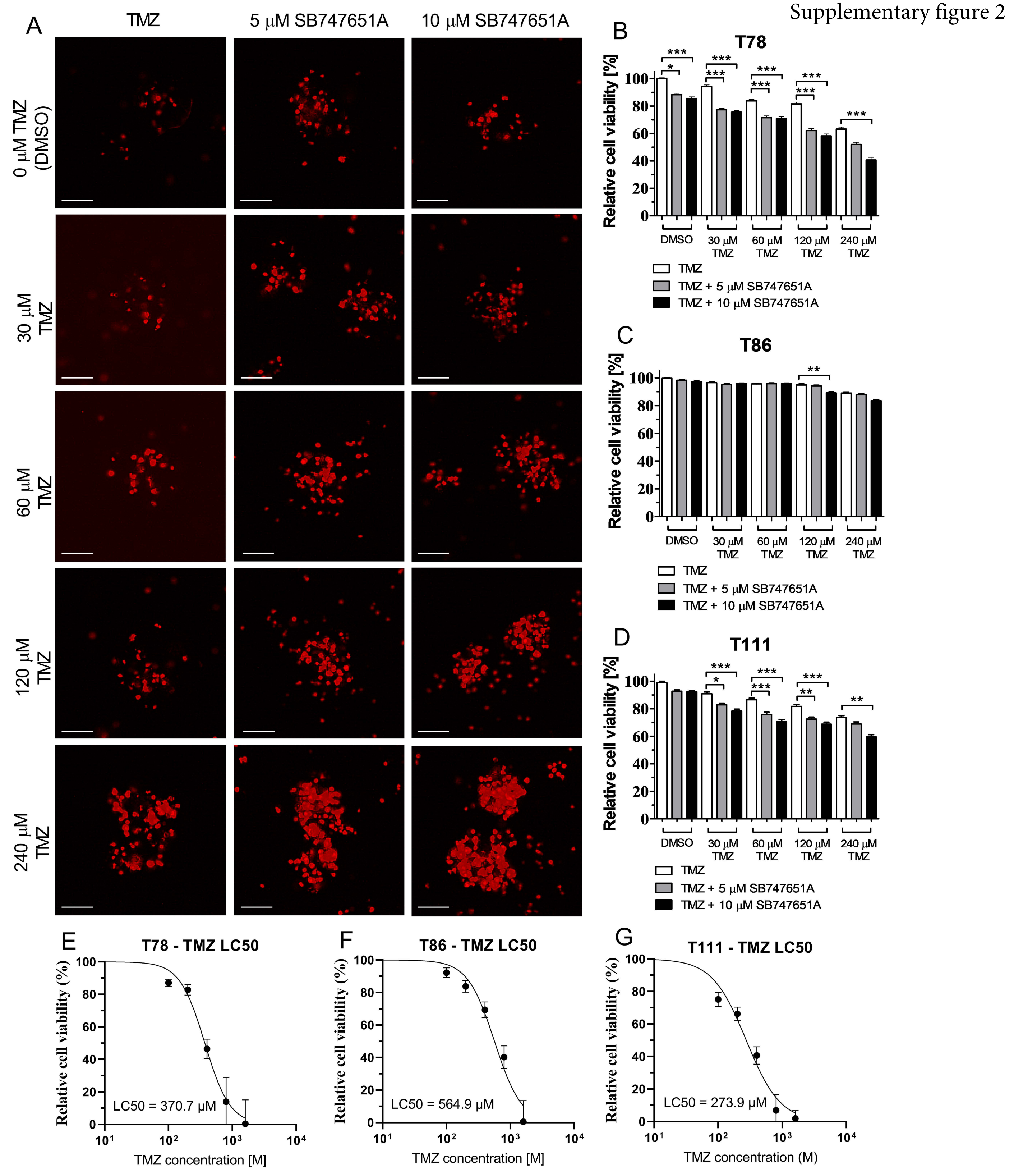

Supplementary Figure 2. TMZ sensitivity assays.

A) Representative fluorescence images of spheroids from the T78 spheroid culture after exposure to combinations of Temozolomide and SB747651A for 48 hours. **B)** The T78 spheroid culture showed a significant concentration-dependent synergistic effect on cell death when combining TMZ and SB747651A. **C)** The T86 spheroid culture was highly resistant to both TMZ and TMZ + SB747651A combinations. **D)** The T111 spheroid culture showed the same concentration-dependent synergistic effect as observed in the T78 spheroid culture. LC50 values following 48 hours TMZ exposure are depicted for the **E)** T78 **F)** T86, and **G)** T111 spheroid cultures. All displayed significance levels are based on comparisons between the TMZ and TMZ + SB747651A groups respectively. Fluorescence intensity is shown as arbitrary units. * = $P<0.05$. ** = $P<0.01$. *** = $P<0.001$. Scale bar = 100 μm.

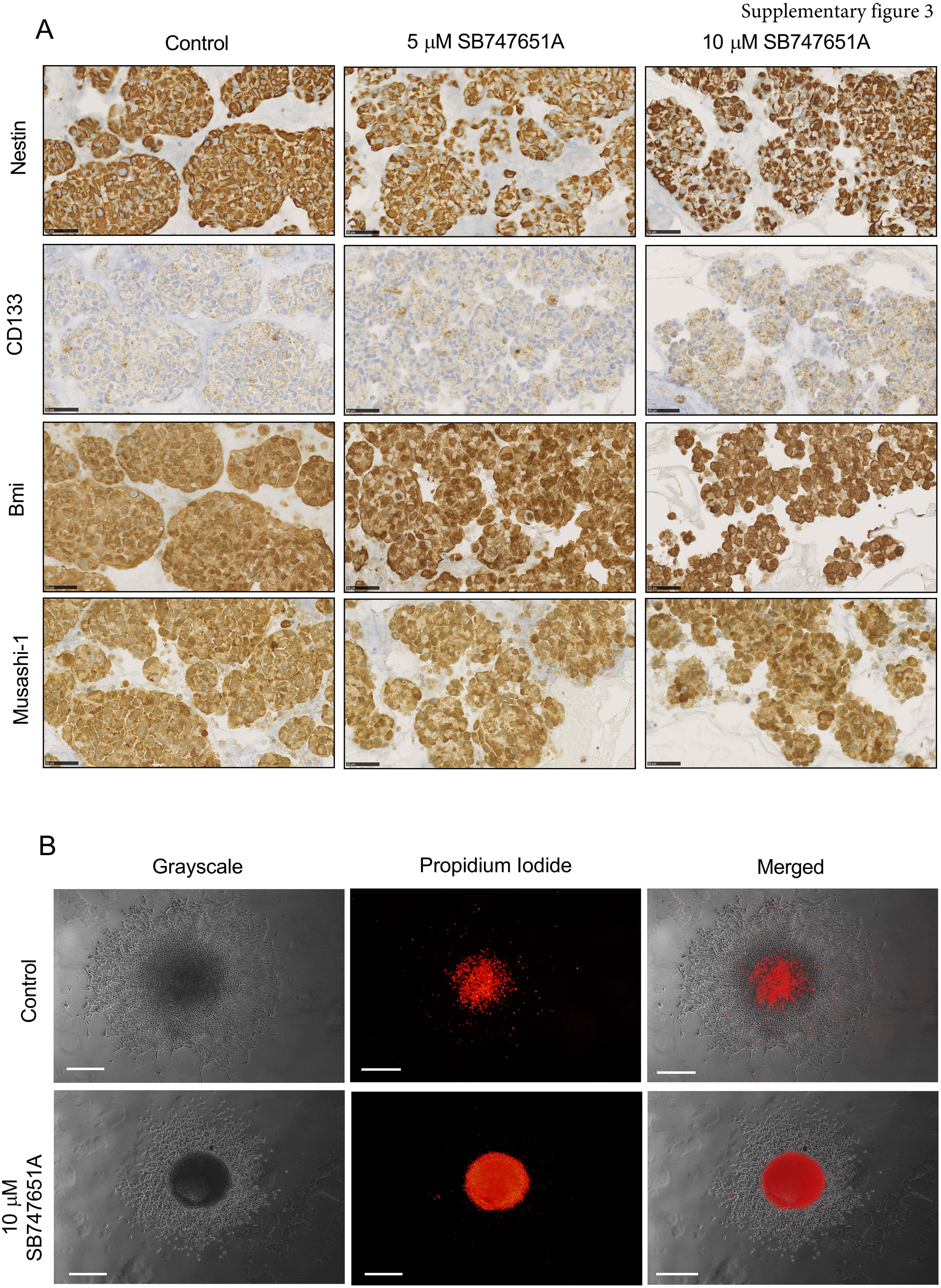
A
Control
5 μM SB747651A
10 μM SB747651A
Nestin
CD133
Bmi
Musashi-1
B
Grayscale
Propidium Iodide
Merged
Control
10 μM SB747651A

Supplementary Figure 3. Immunohistochemical stainings of stemness related markers and supplementary migration assay data. **A)** Immunohistochemical stainings with antibodies against Nestin, CD133, Bmi and Musashi-1 did not show any notable differences in expression levels in SB747651A exposed spheroids vs. controls. Scale bar = 50 µm. **B)** Addition of PI to migrating tumor cells from the migration assay showed that SB747651A did not kill the migrating tumor cells after 72 hours migration. Scale bar = 200 µm.

Supplementary figure 4

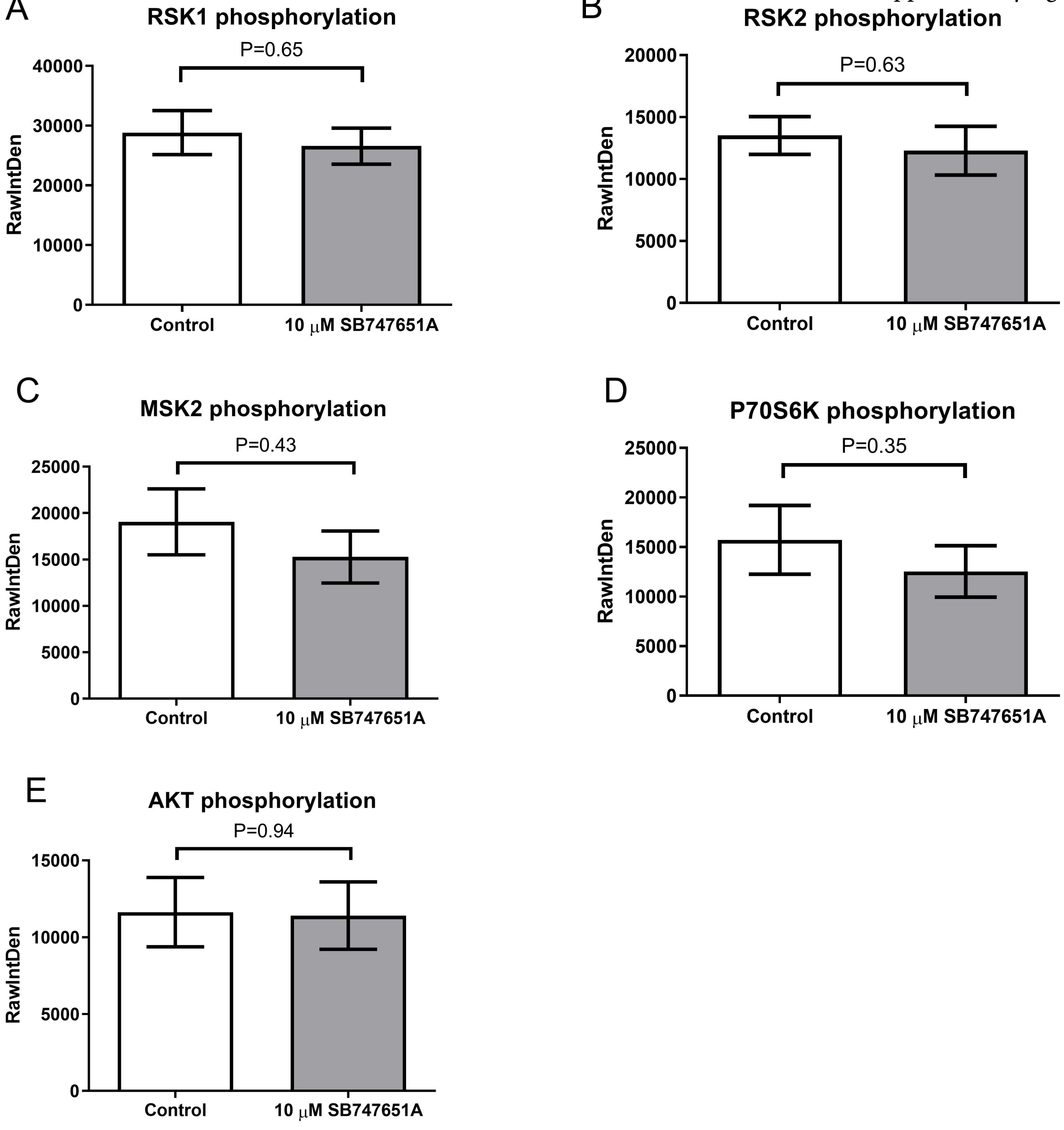

Supplementary Figure 4. Phosphorylation changes of relevant MAP-kinases after SB747651A exposure were measure with the Proteome profiler human phospho-MAPK array kit. Quantification of 4 independent experiments with 72 hours SB747651A exposure showing changes in phosphorylation in **A)** RSK1, **B)** RSK2, **C)** MSK2, **D)** P70S6K and **E)** Akt.

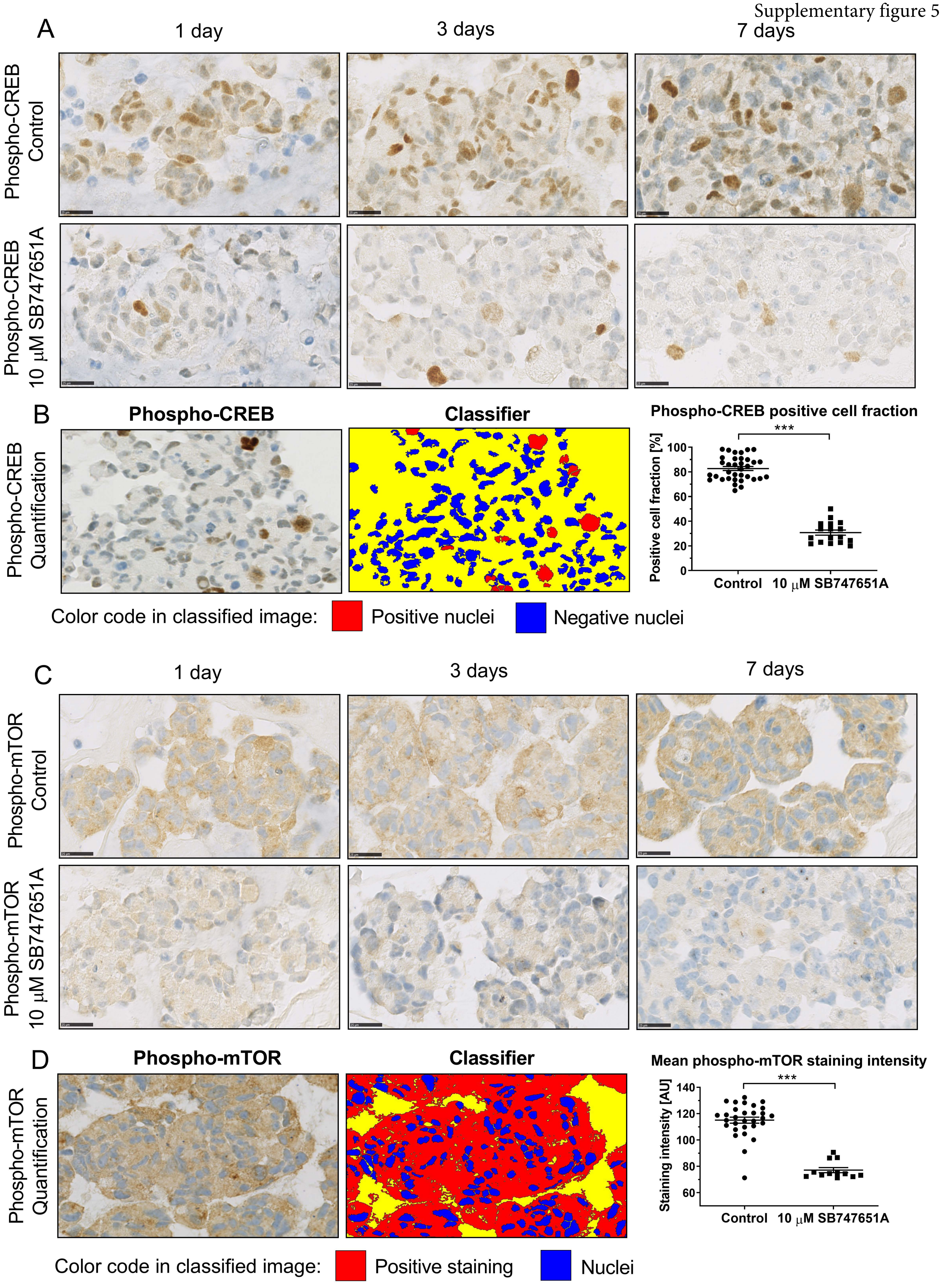
A
1 day
3 days
7 days
Phospho-CREB Control
Phospho-CREB 10 μM SB747651A
B
Phospho-CREB
Classifier
Phospho-CREB positive cell fraction
Phospho-CREB Quantification
Positive cell fraction [%]
***
Control
10 μM SB747651A
Color code in classified image:
Positive nuclei
Negative nuclei
C
1 day
3 days
7 days
Phospho-mTOR Control
Phospho-mTOR 10 μM SB747651A
D
Phospho-mTOR
Classifier
Mean phospho-mTOR staining intensity
Phospho-mTOR Quantification
Staining intensity [AU]
***
Control
10 μM SB747651A
Color code in classified image:
Positive staining
Nuclei

Supplementary Figure 5. Immunohistochemical validation of reduced phospho-CREB and phospho-mTOR levels after SB747651A treatment of spheroids.

A) Representative images of phospho-CREB immunohistochemical stainings at different SB747651A exposure times. **B)** An example of the software-based digital quantification is shown to demonstrate performance of the classifier. Quantification of the positive cell fraction showed a significant reduction of phospho-CREB positive cells in treated vs. control cells. **C)** Representative images of phospho-mTOR immunohistochemical stainings at different SB747651A exposure times. **D)** Quantification of phospho-mTOR staining intensity was also found to decrease significantly following SB747651A exposure compared to controls. AU = arbitrary units. * = $P<0.05$. Scale bar = 50 µm.

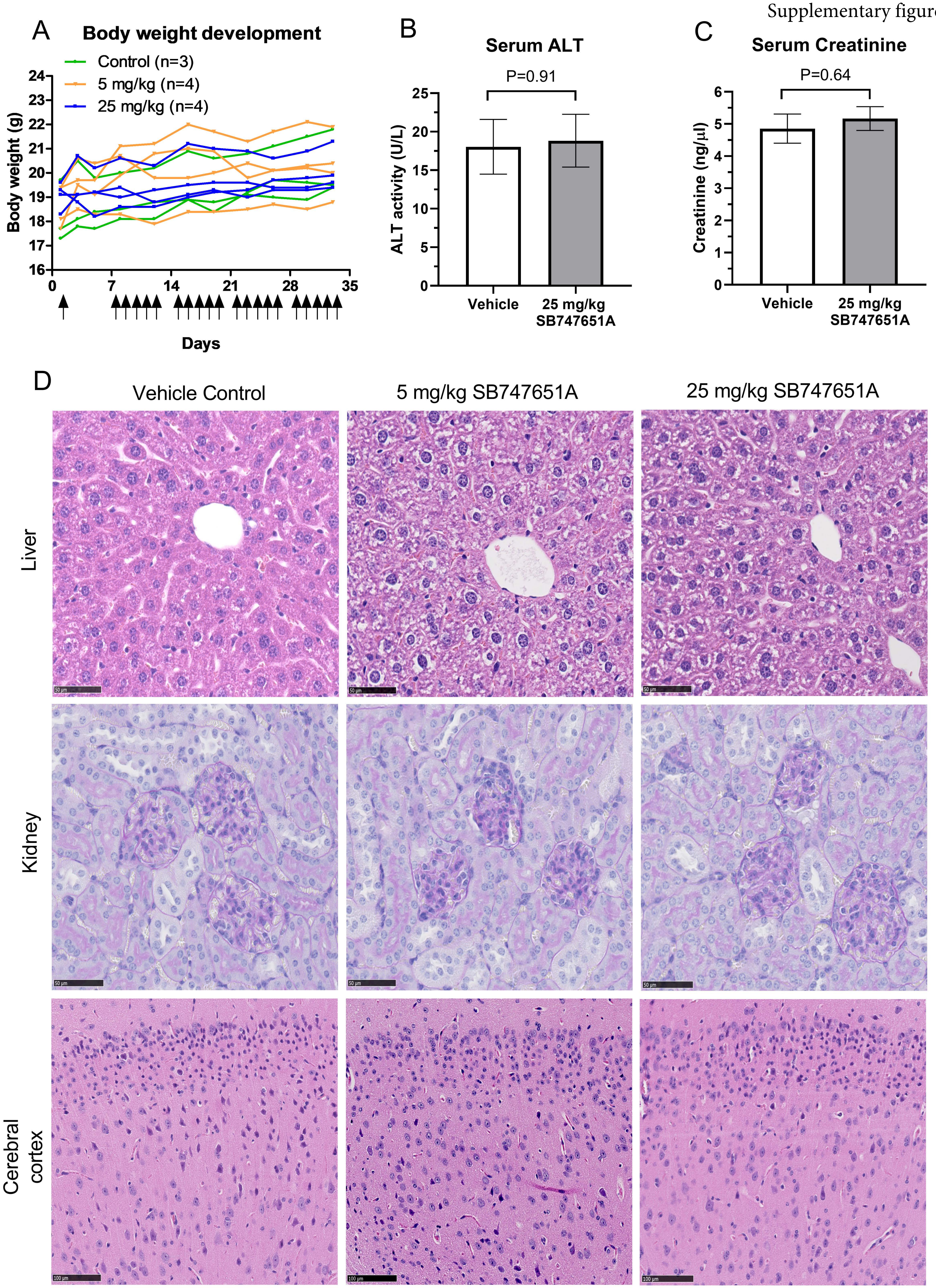
A
Body weight development
Control (n=3)
5 mg/kg (n=4)
25 mg/kg (n=4)
Body weight (g)
24
23
22
21
20
19
18
17
16
0
7
14
21
28
35
Days
B
Serum ALT
P=0.91
ALT activity (U/L)
25
20
15
10
5
0
Vehicle
25 mg/kg SB747651A
C
Serum Creatinine
P=0.64
Creatinine (ng/µl)
6
5
4
3
2
1
0
Vehicle
25 mg/kg SB747651A
D
Vehicle Control
5 mg/kg SB747651A
25 mg/kg SB747651A
Liver
Kidney
Cerebral cortex

Supplementary Figure 6. Basic *in vivo* toxicology screen.

A) Weight monitoring of treated mice showed no weight loss over a 5 week treatment period. Black arrows indicate SB747651A administrations. **B-C)** Measurement of serum Alanine transaminase activity and Creatinine levels in SB747651A and vehicle treated animals (n = 9 per group). **D)** Representative histological sections from organs of mice in the different treatment groups. Liver sections displayed normal architecture and no signs of ductopenia, ductular reaction, inflammation, hemorrhage or necrosis. There was no interphase activity and the lobular parenchyma showed normal sized liver plates. The kidneys showed no signs of inflammation, necrotic cells or degenerative changes. Cerebral cortex had regular cell density and normal nerve- and glial cells. There were no signs of astrogliosis, pyknotic nuclei, cellular damage to purkinje cells or neurons. Scale bar in liver and kidney sections = 50 µm, scale bar in brain sections = 100 µm.
